# One Decade of Environmental Disasters in Brazil: The Action of Veterinary Rescue Teams

**DOI:** 10.3389/fpubh.2021.624975

**Published:** 2021-04-22

**Authors:** Carla Sássi, Gabriel Domingos Carvalho, Leonardo Maggio de Castro, Cláudio Zago Junior, Vânia de Fátima Plaza Nunes, Arthur Augusto Tavares do Nascimento, Ana Liz Ferreira Bastos, Luciana Guimarães Santana, Ilka do Nascimento Gonçalves

**Affiliations:** ^1^G.R.A.D. Grupo de Resgate de Animais em Desastres, Cuiabá, Brazil; ^2^Federal Institute of Education, Science and Technology of Espírito Santo, Ifes Campus Piúma, Piúma, Brazil; ^3^Large Animal Veterinary Hospital, University of Sorocaba, Sorocaba, Brazil; ^4^Fire Brigade School, Military Police of São Paulo State, São Paulo, Brazil; ^5^National Animal Protection and Defense Forum, Jundiaí, Brazil; ^6^G.R.A.D. Grupo de Resgate de Animais em Desastres, Cuiabá, Brazil; ^7^Regional Council of Veterinary Medicine of Minas Gerais—CRMV/MG, Belo Horizonte, Brazil; ^8^Animal Welfare Commission of CRMV/MG, Belo Horizonte, Brazil; ^9^Independent Researcher, Veterinary of Wild Animals, São Paulo, Brazil; ^10^Independent Researcher, Private Veterinary Clinic, Bahia, Brazil

**Keywords:** Brazilian pantanal, Brumadinho, ecohealth, Mariana, one health, planetary health

## Abstract

Based on the interdisciplinary concept of One Health, EcoHealth, and Planetary Health, this paper focuses on participatory knowledge-to-action approaches by relating one decade of environmental disasters in Brazil with the action of veterinary rescue teams, aiming to give support to future disaster preparedness. This paper will present the historic actions of teams rescuing animal that are victims of environmental disasters, in addition to addressing the need for contingency plans and response management in these types of events. The main events in Brazilian states where veterinary rescue teams participated were, chronologically, as follows: 2011 flood and landslide (Rio de Janeiro); 2012 flood (Acre, Minas Gerais, and Pará); 2015 dam break (Minas Gerais); 2017 flood (Minas Gerais) and forest fire (Minas Gerais and Goiás); 2019 dam break and evacuation (Minas Gerais) and flood (Bahia); 2020 flood (Espírito Santo and Minas Gerais) and forest fires (Mato Grosso and Mato Grosso do Sul). The Brazilian disasters that had a large global repercussion were the ruptures of the ore dams in Marina (2015) and Brumadinho (2019), both in the State of Minas Gerais. The role of veterinarians in these events was recognized by the Federal Council of Veterinary Medicine (CFMV) after their performance in Mariana, Minas Gerais (2015), and in 2020, the CFMV approved the National Mass Disaster Contingency Plan Involving Animals. The work of veterinarians in interaction with other professionals in environmental disasters proved to be effective and necessary for the rescue of animals and for planning and giving support to disaster preparedness in the future.

## Introduction

The term “disaster” has different concepts, but the concepts invariably refer to at least one of these factors: threat, vulnerability, risk, exposure, and responsiveness (1). Disasters, regardless of their technical classification, are undesirable and unpredictable events that generate great instabilities in an affected population, negatively impacting directly and indirectly environmental and socioeconomic conditions in a near or distant manner, depending on their magnitude (2).

In general, disasters devastate homes, establishments, and properties, destroying livelihoods and deteriorating essential services, damaging the individual and collective health of humans and animals, reflecting on injured individuals and a variable number of deaths, in addition to leading to damage to a greater and lesser extent and also to mental damage that may be transient or long-lasting (3).

Depending on the type of disaster, different demands are faced by the communities and teams responsible for providing support in the post-tragedy. For this, the professionals of these teams must have multidisciplinary skills from previous training, as well as adequate resources for proper support (4, 5). In some countries, such as the United States, disaster response teams are composed of firefighters, doctors, paramedics, engineers, and machinery operators (tractors and cranes), specialized in rescue, with the duty to locate, extract, and provide assistance (6, 7).

Adverse events, mainly of climatic origin such as droughts and forest fires, of hydrological origin such as runoffs and floods, and of meteorological origin such as heat waves and tropical cyclones, currently affect populations worldwide and particularly in Brazil. According to data from the United Nations International Strategy for Disaster Reduction (UNDRR), more than 200 million people are affected by disasters of different origins every year (1).

This paper will present a brief overview of the historic actions of volunteer veterinarians in rescuing animal victims of environmental disasters, in addition to addressing the need for contingency plans and response management in these types of events.

### Environmental Disasters in Brazil

In Brazil, the occurrences of disasters, especially those of natural origin, coincide with the deterioration of living conditions in cities, with the occurrence of many damages and losses (1). It is estimated that these phenomena aggravate problem situations such as malnutrition, endemic infectious diseases, and accidents due to extreme events. Additional risks to public health must also be considered: excessive demand on health services, water supply problems, and increase in some diseases (8).

The main events with participation of veterinary rescue teams in Brazilian states were, chronologically, as follows: 2011 in Rio de Janeiro (flood and landslide); 2012 in Acre and Pará (flood) and Minas Gerais (flood); 2015 in Minas Gerais (dam break); 2017 in Minas Gerais (flood and forest fire) and Goiás (forest fires); 2019 in Minas Gerais (dam break and evacuation) and Bahia (flood); 2020 in Espírito Santo and Minas Gerais (flood) and in Mato Grosso and Mato Grosso do Sul (Pantanal forest fires) (9) (Table 1).

**Table 1:** Main events with the participation of veterinary rescue teams in the Brazilian states on the last decade, chronologically.

**Year**	**State**	**Cities/areas**	**Type of disaster**
2011	Rio de Janeiro	Nova Friburgo and other municipalities	flood and landslide
2012	Acre	Rio Branco and Brasiléia	flood
	Pará	Santa Cruz do Arari	flood
	Minas Gerais	Tiradentes, Congonhas, Conselheiro Lafaiete, São João del Rei	flood
2015	Minas Gerais	Mariana and other municipalities in the course of river Doce	dam break
2017	Goiás	Alto Paraíso (*Chapada dos Veadeiros*)	forest fire
	Minas Gerais	Rio Casca	flood
		Ouro Branco	forest fire
2019	Minas Gerais	Brumadinho	dam break
		Barão de Cocais	preventive evacuation (risk of dam break)
	Bahia	Coronel João de Sá	dam break
2020	Espírito Santo	Iconha, Rio Novo do Sul, Alfredo Chaves, Cachoeiro de Itapemirim, Castelo, Vargem Alta, and other municipalities	flood
	Minas Gerais	various cities	flood
	Mato Grosso	various cities (*Pantanal*)	forest fire
	Mato Grosso do Sul	various cities (*Pantanal*)	forest fire

In January 2011, heavy rains triggered what would be considered the worst Brazilian natural disaster of recent times: the floods and landslides in the mountain region of Rio de Janeiro, an event that caused 905 deaths in seven cities and affected more than 300,000 people, which corresponded to 42% of the population of the affected municipalities, that is, 4.46% of the population of the State of Rio de Janeiro at the time. Total losses were estimated at US$3 billion; however, these omit relevant impacts in sectors such as education and health, which could not be considered due to unavailability of detailed information (1).

The national disasters, in Brazil, that had a large global repercussion were the ruptures of ore dams in Marina (2015) and Brumadinho (2019), both in the State of Minas Gerais, with is the state with highest number of disasters and consequentially rescue team actions (10). In the case of the Mariana disaster, even other states had been affected, for example, the State of Espírito Santo, where the mouth of the river Doce, sourced in Minas Gerais, is located. Some of these impacts were observed in estuarine fish, through tissue bioaccumulation and oxidative stress defenses observed in response to the contamination of the river Doce (11).

The most recent disaster in Brazil is the Pantanal fire in the States of Mato Grosso and Mato Grosso do Sul. In 2020, it is estimated that the fire destroyed 28% of the Brazilian Pantanal between January and October, as monitored by the Environmental Satellite Applications Laboratory at the Federal University of Rio de Janeiro (*Laboratório de Aplicações de Satélites Ambientais da Universidade Federal do Rio de Janeiro* [LASA/UFRJ]). Burnt areas increased by more than 100%, compared to the same period at the year previous to the disaster. Fire affected almost all conservation units and indigenous lands in the Pantanal region (12).

### Disasters and Environmental, Human, and Animal Health

Disasters result in direct short-, medium-, and long-term effects as well as indirect effects on the health and well-being of populations. Among these effects, the following stand out: reduction of social welfare standards; deaths, traumas, and injuries; damage to the basic service structure; compromise of equipment and medicine stocks; proliferation of infectious and vector-borne diseases; and psychosocial damages (8). The most health-related problems involve the same complex, humans–animals and the environment, so government decision-making should be based on the pillars of the One Health concept, based on the knowledge produced and interconnected by different institutions, based on the problems found in society, acting from an intersectoral and multiprofessional perspective (13).

The measures adopted in veterinary medicine during disasters are based on the same pillars used for the human population; however, it is necessary to adjust them to the specific needs of different species (10). In Table 2 are presented the main actions done by the rescue veterinary teams in case of disasters. Some rescue situations are high-risk scenarios and difficult for rescuers to access, as in the case of Brumadinho, which leads those responsible for rescuing the animals to adopt extreme measures but always backed by an ethical professional attitude (14).

**Table 2:** Main actions of rescue veterinary teams in cases of disasters.

**Veterinary actions in animal rescues during disasters**		
**Before rescue**	**During rescue action**	**After rescue**
1) survey of previous information of the affected areas (accesses, topography, activities developed in the place, type of residences, and others)	1) rescue of survivors	1) clinical follow-up of survivors
2) identification of the main animal species in the region	2) clinical and surgical care	2) transfer rescued animals to guardians or adopters
3) elaboration of the action plan	3) shelter installation	3) castration of the animals that will be sent for adoption
4) organization of first-aid work material (personal protective equipment, medicines, hospital supplies)	4) vaccination protocols and application of antiparasitic drugs	4) serological diagnosis for epidemiological investigation of endemic diseases in the affected region
5) water demand for survivors	5) providing water and food to survivors	5) Monitoring of zoonoses

The diagnosis of the situation of a specific affected area is of paramount importance to identify the main basic needs of the population in the region (15). Previous knowledge about the region and the database of the municipalities are essential to understand what activities are carried out there and the estimated number of properties, residents, and animals in the region. Sanitary and animal health data are important as a starting point for the formulation of specific epidemiological indicators (6, 10, 16).

### Action of Veterinarians in Animal Rescues During Disasters

In 2011, with the catastrophe caused by heavy rains and landslides that had hit the mountainous region of the State of Rio de Janeiro, Brazil, in the city of Nova Friburgo, the first team of veterinary medical professionals, originating from the State of Minas Gerais, started to voluntarily train themselves to act in rescues of domestic animals in situations of environmental disasters (9).

Over the years, these groups realized the need for training so that their work has become increasingly technical and successful. The collective veterinary medicine is of great importance in this scenario and training, for having been a pioneer in addressing the theme of mass disasters and its impact on the lives of animals in Brazil. It is essential that professionals get deeper into collective veterinary medicine to act during mass disasters, since the area encompasses many aspects inherent to crisis scenarios, such as animal welfare, zoonoses, animal behavior, adoption, bioethics, human resource management, and even humanitarian education, balancing in these interfaces the elements that constitute unique health (9). In addition to these aspects, it is essential to train the teams and provide technical and psychological support (10).

Since then, the need to formalize and structure veterinary rescue groups arose. The Disaster Animal Rescue Group (*Grupo de Resgate de Animais em Desastres* [GRAD]) was officially created in 2019. Before the dam burst in Brumadinho, several professionals and volunteers that operate in disasters were included in the group. Important work fronts were developed and technologized, such as contingency plans, autopsies, vaccination and health of the team, and veterinary medicine from catastrophe animal shelters, in addition to the field rescue fronts. With the constant performance of veterinary professionals in catastrophes and with the insertion of a team in the Regional Council of Veterinary Medicine of Minas Gerais, a new line of professional activity originated, the veterinary medicine of disasters (17).

GRAD is a group constituting veterinarians and volunteers, which is acknowledged nationally by the National Animal Protection and Defense Forum (FNPDA), receiving support from civil society and from the Federal Council of Veterinary Medicine (CFMV), being nationally recognized for its experience in response to fauna affected by disasters (17). The main disasters where GRAD members had participated are listed in Table 1.

In view of this scenario of the last decade, there is a clear need to prepare veterinarians to work in this area, so that they can be recognized, respected, and inserted in an official way in disaster management operations, acting in parallel with the actions developed for the rescue of human lives. It is essential to recognize the role of animals in the family nucleus and human health and include them in disaster contingency plans, with a view to preventing and reducing the health risks arising from these disasters (18). Biosafety measures during disasters should be part of the operation and management plan to prevent anthropozoonoses, as they are essential for the well-being and health of humans, animals, and the environment, ensuring One Health (9).

### National Mass Disaster Contingency Plan Involving Animals

In mass disasters, it is essential that there is articulation between several federal, state, and municipal institutions that can contribute to situations involving animals, such as environment, agriculture, public health, police, public ministry, civil defense, firefighter, education, and civil society organizations. It is essential that contacts and partnerships between institutions and support bodies begin even before the occurrence of a disaster, to harmonize contingency strategies and plans and to promptly implement them, correctly and at the right time (9). There is a need for information and surveillance systems integrated between areas such as public health, civil defense, and environmental defense to analyze the effects of disasters on the health of populations (19).

In Brazil, the National Civil Protection and Defense Policy (PNPDEC), established through Law 12608/2012, provides that civil defense and protection actions are organized by prevention, mitigation, preparation, response, and recovery actions. Thus, for each of them, there are specific responsibilities, while they are part of a systemic and continuous management. However, this policy does not detail the actions related to animals as among the main actions to assist victims of disasters; it mentions only the management of domestic animals and the burial of animals in appropriate places, according to zoonosis rules (1), not considering the whole concept of One Heath.

The creation of action plans for animal rescue in environmental disasters requires good strategic planning and investment of resources, participation of public authorities, qualified training of the professionals involved, and the awareness of the population about basic preventive actions (20). The groups and official bodies that deal with mass disasters in Brazil, in general, do not have the participation of professionals specialized in the care of animals. Thus, veterinary medical professionals have been assuming this role on a voluntary basis for some years (9).

In October 2020, the CFMV of Brazil approved the National Mass Disaster Contingency Plan Involving Animals to support the conduct of professionals working in the field (21). The document provides guidelines for the performance of professionals in scenarios of this nature, with guidelines on how to conduct rescue, veterinary assistance, maintenance, and disposal of domestic and wild animals. The plan is a milestone and has become the reference for professionals working in all states of the country (22).

The plan is the result of the CFMV's Mass Disaster Involving Animals Working Group, which was attended by members of GRAD, to support actions in the response and prevention of the next disasters, which generate impacts for society, with implications for public health, the economy, and the emotions of the affected population, especially of animals that are vulnerable, be they companionship, production, or wild animals. According to the CFMV, the construction of the plan was only possible by observing and documenting the difficulties faced in national disasters that have occurred since 2011, with the floods and landslides of Nova Friburgo, in Rio de Janeiro; the ruptures of dams in Mariana (2015) and Brumadinho (2019), in Minas Gerais; and the fires in the Pantanal (2020) (23).

The National Contingency Plan for Mass Disasters Involving Animals brings together the experience of professionals from various segments of activity, considering the particularities of the species and the potential disasters expected for Brazil. The plan considered aspects of approaching the scenario and making decisions involving the care and rescue of the various species; their habits, food, accommodation, transportation, and health; and all the spheres that need to be understood so that prevention, response, and/or recovery are successful, ensuring the rescue of animals and guaranteeing their well-being and quality of life, with clear and concise guidelines. It is believed that the plan will be an important marker of activities related to the theme in future situations, which cannot be predicted, but for which one must be prepared (9).

Technical preparation, hierarchy, and communication in the context of a disaster are essential for the safety of professionals, people, and animals, as well as for planning and decision making. However, to make effective and assertive conducts feasible in handling these situations, the accurate compilation of information and data by the situation diagnosis team is vital, helping to mobilize efforts and adequate resources for the operation (17). Previous planning deserves emphasis and gains great prominence for the positive execution of operations, which is sometimes more important and effective than frontline actions in the field. The alignment of disaster veterinary medicine professionals with official municipal, state, and federal agencies is essential for the success of rescue actions (24).

Technical rescue of animals in disaster scenarios involves planning and, at the same time, requires speed. To facilitate the conduct of professionals, the National Mass Disaster Contingency Plan Involving Animals highlights eight steps to be observed, aiming at the health and well-being of animals and specifying plans to rescue and welcome oxen, horses, pigs, rabbits, dogs, cats, birds, fish, and domestic rodents. It involves everything from on-site assistance, with water, food, medication, and animal preparation (some even require sedation), to transportation and disembarkation at the destination, in temporary shelters. In the operational part, in addition to providing guidance on initial diagnosis, action plans, composition, and team meetings, the plan also defines priorities and strategies for assisting animals. The document addresses cases subject to euthanasia provided for in legislation and guides the conduct of crime scene investigations, which includes collecting corpses and biological and chemical remains, preserving the chain of custody, and maintaining the suitability of the remains from their recognition and collection until its use by the justice department as an evidence element (23).

The plan also addresses aspects pertinent to legal veterinary medicine, forensic necropsy, biosafety measures and personal protective equipment, immunization of workers and volunteers, health service waste management plan, and work zones. It also deals with a hierarchical structure for organizing the responsibilities of official bodies and their actions during the response to a disaster. The plan also describes what the documentation system for veterinary medical care should be in the routine of temporary shelters for rescued animals and indicates how to deal with the destination of domestic animals for temporary home, adoption, or reintegration with the guardian (22).

## Discussion

Like people, animals are also victims of disasters, and they need to receive due attention, following ethical, legal, sanitary, social, and environmental protocols. Over the course of one decade, rescue efforts were improved, and rescue techniques and procedures were developed for different animal species in different types of catastrophe situations, as well as standard operating protocols, first-aid protocols in the field, protocols for use of anesthetics in the field, and vaccine and medication protocols for each type of species affected, in addition to training teams to work on different fronts.

The disaster veterinary medicine is an emerging area with a strong humanitarian bias and requires social motivation because there are several situations that professionals face in these occurrences that require preparation and continuous training. In addition, emotional intelligence is needed to face the realities encountered in disaster situations, such as environmental destruction, extreme contexts of crisis, dangerous situations, and vulnerability involving the homeless, missing, and dead. In times of disaster, the teams involved are faced with a chaotic and complex environment, which requires coordinated and integrated action by multiple agencies, aimed at mitigating suffering and damage.

Another point to be highlighted is that after the occurrence of a series of disasters and the work of voluntary rescue teams, there has been a greater appreciation of the veterinary medicine class by society and companies, especially companies that provide services for the systemic monitoring of fauna in affected areas and are thus considering hiring veterinarians to be integrated into multidisciplinary teams.

The environmental disasters that occurred in Brazil showed the importance of professionals that act in disasters. In contexts such as large fires, landslides, floods, ruptures of tailings dams, and natural disasters (such as tornadoes and storms), veterinarians work mainly in the rescue and clinical and surgical care of animals of different species of domestic and wild fauna. However, these professionals can also form activities in the field of food security for the affected population, in pest control, and, in action planning, in integrated work of several teams involved in the affected regions, which emphasizes the interdisciplinary profile of the veterinary medicine in the concept of One Health.

The work of veterinarians in interaction with other professionals in environmental disasters proved to be effective and necessary for the rescue of animals, not only because they are part of the affected families and because animals are sentient beings but also because they are important characters in the epidemiological scenario before, during, and after a disaster has occurred. We consider that this information is essential to influence scientific priorities in this approach and give support nationally and internationally for public policies and decision-making at local and global levels for disaster preparedness in the future with a focus on the approach of EcoHealth and Planetary Health.

With the content of the National Mass Disaster Contingency Plan Involving Animals, in Brazil, it is expected that the actions of rescuing animals in situations of mass disasters can be officially recognized and incorporated into the activities of the agencies and institutions responsible for responding to crisis scenarios.

## Author Contributions

CS and GC contributed to the conception and design of the paper and organized the information of the presented data. GC organized the paper text. All authors revised and contributed with their professional and personal experience in the actions on the rescue of animals in the environmental disasters in Brazil, in different regions and years.

## Conflict of Interest

The authors declare that the research was conducted in the absence of any commercial or financial relationships that could be construed as a potential conflict of interest.
